# *Candida utilis* as an emerging rare cause of septicemia among neonates and children in Western Uttar Pradesh: A case series

**DOI:** 10.22034/cmm.2024.345218.1529

**Published:** 2024-05-07

**Authors:** Dharmendra Prasad Singh, Rajesh Kumar Verma, Rajesh Kumar Yadav, Ketan Anant, Anupam Das

**Affiliations:** 1 Department of Microbiology, Uttar Pradesh University of Medical Sciences, Etawah, India; 2 Department of Paediatrics, Uttar Pradesh University of Medical Sciences, Etawah, India; 3 Department of Microbiology, Dr. Ram Manohar Lohia Institute of Medical Sciences, Lucknow, India; # Equally contribution

**Keywords:** Antifungal susceptibility, *Candida utilis*, MALDI –TOF MS, Septicemia, VITEK® 2, Pediatric

## Abstract

**Background and Purpose:**

*Candida utilis* is a recently emerging nosocomial fungal pathogen. Candidemia is the fourth most prevalent cause of bloodstream Infections with mortality rates varying from 5-71%.

**Materials and Methods:**

This was a retrospective study conducted at Uttar Pradesh University of Medical Sciences, Etawah, India, from September 2023 to February 2024. Rapid identification was performed by VITEK® 2 (BioMérieux, France) and 18 out of 20 *C. utilis* cases were verified by matrix assisted laser desorption ionization-time of flight mass spectrometry (BioMerieux, France). Susceptibility testing was conducted by VITEK® 2 appropriate Card.

**Results:**

*Candida utilis* was mainly observed between 0-9-month-old neonates, except one case of 11 years old. The extended Intensive Care Unit stay and prior antibiotic use were common risk factors in all cases. They were pan susceptible to each of the tested antifungal medications, and 6 out of 10 cases showed positive clinical response after antifungal treatment.

**Conclusion:**

Early identification and prompt treatment favors a good clinical outcome. The current research primarily aimed to elaborate on the speciation, incidence, and antifungal susceptibility testing of *C. utilis* at a tertiary care center.

## Introduction

*Candida* species is becoming a significant factor in causing sepsis in critically sick patients, leading to considerable mortality and morbidity, and contributing to 10-15% of healthcare-related infections [ 1 ]. Prevalence rate of bloodstream infection (BSI) in neonates is 9-13% [ 2 ], with a notable percentage of these infections having nosocomial origins. In India, the incidence of candidemia is 6-18%, with a rising trend of non-*albicans Candida*‎ (NAC)‎ species‎ causing BSI. An analysis conducted in Western India found a 2.8% occurrence of candidemia along with ‎NAC ‎ species ‎ making up 89% of the cases [ 3 ]. *Candida utilis* is commonly used in food industry as a yeast additive and has relatively low virulence, compared to other pathogenic *Candida* spp., but is increasingly a recognized cause of candidemia in newborns. *Candida utilis* catheter-associated blood stream fungemia has mainly been reported in immunocompromised patients as well as in neonates [ 4 , 5 ].

Previous case studies and investigations indicate a low death rate of *C. utilis* and its susceptibility to every tested antifungal medication [ 5 ]. VITEK® 2 provides a species identification and susceptibility pattern of antifungal drugs. Matrix-assisted laser desorption–ionization-time of flight mass spectrometry (MALDI-TOF MS) can quickly detect and differentiate yeasts in blood culture samples [ 6 ]. 

In the case of recently developing pathogens, systematic monitoring by advanced automated systems is used to identify rare emerging pathogens of candidemia and antifungal susceptibility profile, particularly in neonates. It will be helpful in timely diagnosis and prompt treatment of candidemia [ 7 , 8 ]. 

## Materials and Methods

The retrospective case series was conducted at a tertiary care center. The clinical and demographic information of every patient with *C. utilis* isolated from blood cultures at the Department of Clinical Microbiology between September 2023 and February 2024 was examined by an electronic review of patient records. According to the standard protocol, most samples were inoculated into BD BACTECTM culture vials (Becton, Dickinson and Company, USA) and some from conventional blood culture bottles. BACTEC Blood culture that flagged positive and conventional blood culture plate which show dry white or creamy growth had a gram-stain if yeast-like organisms were seen under a Microscope. *Candida* species identification was conducted by VITEK® 2 YST (BioMérieux, France), Hi-CHROM agar (HiMedia, India) *Candida*, and confirmed using MALDI-TOF MS (BioMerieux, Version 3.2, France). Susceptibility testing was conducted by the VITEK® 2 AST-YS08 Card (BioMérieux, France).

## Results

In this study, 18 isolated cases of *C. utilis* were identified, 10 of which were critically ill to cause septicemia. Summarized demographic characteristics, clinical data, and diagnostic criteria of *C. utilis* cases are presented in Table 1. *Candida utilis* was mainly observed among 0-9-month-old neonates, except one case of 11 years old. The most common diagnoses were sepsis (3 cases), respiratory distress/pneumonia (4 cases), and respiratory failure/shock (2 cases). Two patients had underlying acyanotic congenital heart disease, and one patient had multiple jejuno ileal fistulas as comorbidities. Concomitant infections were noted in 2 patients, with one testing positive for respiratory syncytial virus (RSV). 

**Table 1:** Summarization of *Candida utilis* cases

no.	Age	Final Diagnosis	Comorbidities	ConcomitantInfection	Treatment	Outcome	SNCU/NICU/PICU stay
1	NB	Neonatal Sepsis	Nil	Nil	FLC IV	Stable at discharge	Yes
2	11 Years	Sepsis	Multiple jejuno- ileal fistula	Nil	Amp B + 5FC IV	Died	No
3	7 Days	Severe Pneumonia, Respiratory failure, shock	Nil	Nil	Amp B + 5FC IV	Died	Yes
4	NB	Respiratory distress	Nil	Nil	FLC IV	Stable at Discharge	Yes
5	NB	LBW, Non-Vigorous Meconium-Stained Liquor, Sepsis	Nil	Nil	FLC IV	Stable at Discharge	Yes
6	4 Months	Pneumonia, Acyanotic congenital heart disease	Nil	RSV Positive, Throat swab	FLC IV	Stable at Discharge	Yes
7	9 Months	Respiratory failure, Shock	Nil	Nil	Amp B+ 5FC IV	Died	Yes
8	3 Weeks	Pneumonia, Acyanotic congenital heart disease	Nil	Nil	Amp B+ 5FC IV	Stable at discharge	Yes
9	16 Days	Severe sepsis, Shock LBW	Nil	Nil	FLC IV	Died	Yes
10	2 Months	Pneumonia, Sepsis	Nil	Nil	FLC IV	Stable at discharge	Yes

FLC- Fluconazole, NB- Newborn, LBW- Low Birth Weight, Amp-B- Amphotericin-B, 5FC-Flucytosine, IV- intravenous (IV) injection, RSV-Respiratory syncytial virus

Regarding antifungal treatment, seven patients received fluconazole, and three patients received a combination of amphotericin B and flucytosine. The outcomes varied, with 6 patients showing stability at discharge, while four patients unfortunately succumbed to their illnesses. All patients required a stay in the Special newborn care unit, neonatal intensive care unit, or pediatric intensive care unit during their treatment. The extended intensive care unit stay and prior antibiotic use were common risk factors in all cases. 

Methods of identification and susceptibility pattern results are presented in Table 2. All 10 isolates were successfully identified as *C. utilis* by both MALDI-TOF MS and VITEK® 2 YST, demonstrating concordant results. The isolates exhibited pan-susceptibility to all tested antifungal agents. Antifungal susceptibility testing revealed that all isolates were susceptible to fluconazole (MIC range: ≤0.12 to 2 μg/mL) and voriconazole (MIC range: ≤0.12 to 0.5 μg/mL), with interpretative readings of susceptible. For amphotericin B, all isolates had MIC values ranging from 0.25 to 0.5 μg/mL, which were also interpreted as susceptible. Regarding flucytosine, all isolates were susceptible, with MIC values ranging from ≤1 to 4 μg/mL.

**Table 2:** Methods of identification and susceptibility pattern used in this study

Identification methods	MALDI-TOF MS Version 3.2	VITEK® 2 YST	VITEK® 2 AST- YS08							
			Fluconazole		Voriconazole		Amphotericin		Flucytosine	
			MIC	INT	MIC	INT	MIC	INT	MIC	INT
Case 1	*C. utilis*	*C. utilis*	1	S	≤0.12	S	0.5	S	4	S
Case 2	*C. utilis*	*C. utilis*	1	S	≤0.12	S	0.5	S	≤1	S
Case 3	*C. utilis*	*C. utilis*	1	S	≤0.12	S	0.5	S	4	S
Case 4	*C. utilis*	*C. utilis*	1	S	≤0.12	S	0.5	S	4	S
Case 5	*C. utilis*	*C. utilis*	2	S	≤0.12	S	0.5	S	≤1	S
Case 6	*C. utilis*	*C. utilis*	2	S	≤0.12	S	0.5	S	≤1	S
Case 7	*C. utilis*	*C. utilis*	2	S	≤0.12	S	≤0.25	S	≤1	S
Case 8	*C. utilis*	*C. utilis*	2	S	≤0.12	S	≤0.25	S	≤1	S
Case 9	*C. utilis*	*C. utilis*	2	S	≤0.12	S	0.5	S	≤1	S
Case 10	*C. utilis*	*C. utilis*	1	S	≤0.12	S	≤0.25	S	≤1	S

*C. utilis*- *Candida Utilis*, MIC- Minimum Inhibitory Concentration, INT- Interpretation

## Discussion

The present retrospective study was conducted in the department of microbiology at Uttar Pradesh University of Medical Sciences in Etawah, India, from September 2023 to February 2024. During the study period, among the *Candida isolates*, non-*albicans Candida* (NAC) spp. (55 out of 64) was found to be predominant. *Candida* utilis is the most common predominant NAC spp. isolated from blood culture. 

Although *C. utilis* is considered a low-virulence pathogen and is rarely isolated from clinical specimens, it is usually used in food industries for nonethanolic fermentation purposes [ 9 ]. However, nowadays, *C. utilis* is infrequently reported from different parts of the world as an opportunistic pathogen for neonatal septicemia [ 4 ]. VITEK^®^ 2 identified 20 *C. utilis* and 18 cases were confirmed by MALDI-TOF MS which accounted for 32% (Table 2). 

Most patients were aged between 0-9 months, except one patient (11 years old). They had history of lethargy, poor feeding, high-grade fever, shortness of breath, and prior hospitalization (in some cases). The most frequent predisposing factors were intensive care stay and prior use of antimicrobials. The same risk factors have been reported in previous *C. utilis* studies [ 8 , 10 ]. Two cases had congenital heart disease with lung infection and eight cases had breathing issues. A concomitant infection of RSV was found in 1 case (Table 1). Antifungal susceptibility testing was performed by VITEK® 2 AST-YS08 card (Table 2) which showed pan-susceptible *C. utilis* strain to fluconazole, voriconazole, amphotericin B, and flucytosine. It also showed MIC values corresponding to four drug concentrations examined, which is very comparable to previous studies [ 6 , 11 ]. To the best of our knowledge and research, this is the first case series of *C. utilis* candidemia in Northern India. In this study, *C. utilis* was found to be the most predominant NAC spp. and emerging as an opportunistic fungal pathogen for neonatal septicemia. Neonatal septicemia is a very serious condition and its prognosis worsens with every passing hour. Therefore, it needs early and rapid identification and prompt treatment. Conventional methods are difficult to identify some NAC spp., such as *C. utilis*. For early identification and antifungal susceptibility testing‎, VITEK® 2 automated techniques are a very promising option and for confirmation molecular characterization or protein profiling, MALDI-TOF MS is currently recommended by World Health Organization [ 12 , 13 ].

## Conclusion

This study underscores the critical importance of early identification and prompt treatment of *C. utilis* infections for achieving favorable clinical outcomes. Our comprehensive analysis of *C. utilis* at a tertiary care center has provided valuable insights into its speciation, incidence rates, and antifungal susceptibility profiles. These findings not only contribute to the existing body of knowledge on *C. utilis* but also offer practical implications for clinical management. By elucidating the prevalence and antifungal resistance patterns of this fungal species, our research equips healthcare professionals with essential information to guide diagnostic and therapeutic decisions, potentially improving patient care and treatment efficacy in tertiary healthcare settings.
